# Supplementary material for: Thiol-Substituted Poly(2-oxazoline)s with Photolabile Protecting Groups—Tandem Network Formation by Light
Source: Polymers (Basel). 2020 Aug 7;12(8):1767. doi: 10.3390/polym12081767 (PMC7463461; doi:10.3390/polym12081767)
Supplement: Supplementary file 1 [file polymers-12-01767-s001.pdf]

Supporting Information

Thiol-Substituted Poly(2-Oxazoline)s with Photolabile Protecting Groups - Tandem Network Formation by Light

*Niklas Jung, Fiona Diehl, Ulrich Jonas**

Macromolecular Chemistry, University of Siegen, Adolf-Reichwein Strasse 2, 57076 Siegen, Germany

*corresponding author Email address: jonas@chemie.uni-siegen.de

---

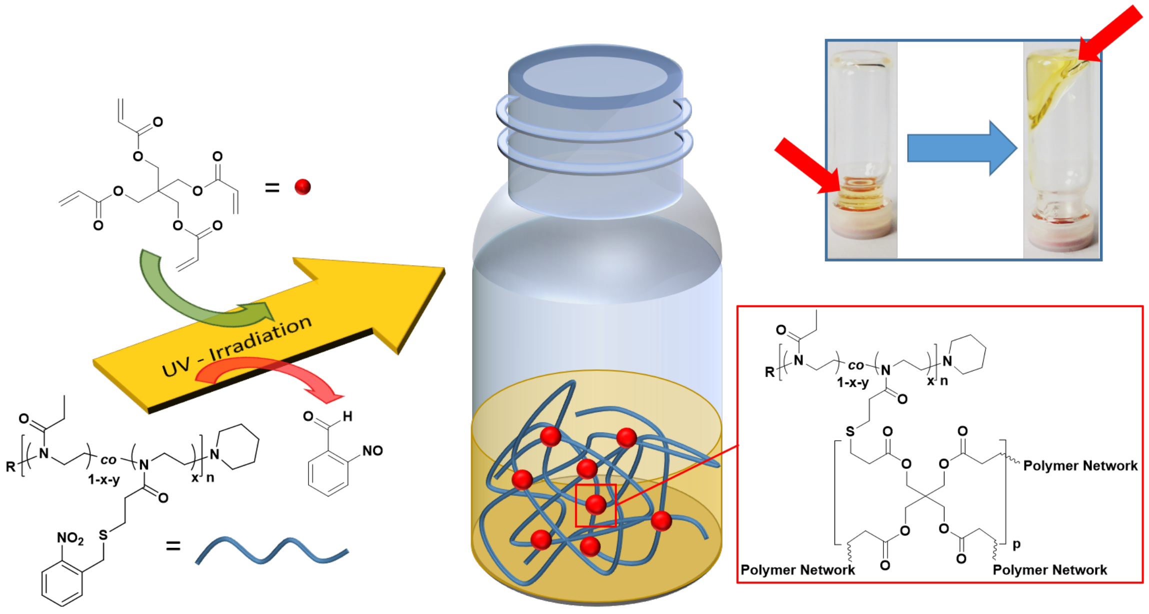

Schematic representation of tandem network formation by concurrent photodeprotection and thiol-ene click coupling (red arrows indicate liquid and gel, respectively).

Table of Content

Experimental Part 1

Synthesis of 2-{2-[(2-Nitrobenzyl)thio]ethyl}-4,5-dihydrooxazole (NbMEtOx) 2

Synthesis of CH_3-Poly[(2-ethyl-2-Oxazoline)$_{1-x}$-co-(NbMEtOx)$_x$]-piperidine (Px%)............. 5

Gelformation using Pentaerythritol tetraacrylate (PETA)... 6

Attempts to synthesize a NbMEtOxa Homopolymer... 6

NMR Spectra of NbMEtOxa and Precursors 8

NMR Spectra of Polymers before and after Deprotection 11

GPC Data of Polymers.. 15

UV/Vis Spectra of Polymers.. 16

Gel Formation 21

Experimental Part

Materials

Acetone (VWR Chemicals, 99.8%) was distilled before use. Further, 2-ethyl-2-oxazoline (EtOxa, Alfa Aesar 99%), methyl trifluoromethanesulfonate (MeOTf, Alfa Aesar, 97%), triethylamine (NEt_3, ChemSolute, 99%), and 2-methyl-2oxazoline (MeOxa, Sigma Aldrich, 98%) were dried over CaH_2 and distilled under inert gas or vacuum prior to use. For polymerizations anhydrous acetonitrile (MeCN, VWR, max. 0.001% H_2O) and for further experiments acetonitrile (MeCN, VWR Chemicals, 99+%) was used. Deuterated solvents were purchased from Deutero GmbH. 2-Chloroethylamine hydrochloride (Alfa Aesar, 98+%), dichloromethane (DCM, Fisher Scientific, 99.8%), diethyl ether (Acros Organics, 99.5%), *N,N*-dimethylformamide (DMF, Carl Roth, 99.8%), 1,4-dioxane (Carl Roth, 99.5%), ethyl acetate (EtOAc, VWR Chemicals, 99.8%), 1-ethyl-3-(3-dimethylaminopropyl)carbodiimide hydrochloride (EDC-HCl, Carl Roth, 99+%), hexanes (Fisher Scientific), hydrochloric acid (VWR Chemicals, 37%), 2-hydroxy-2-methylpropiophenone (HMPP, CIBA, 97%), *N*-hydroxysuccinimide (NHS, Alfa Aesar, 98+%), 3-mercaptopropionic acid (Alfa Aesar, 99%), magnesium sulfate hexahydrate (Carl Roth, 99+%), methanol (MeOH, Fisher Scientific, 99.8%), 2-nitrobenzyl bromide (Alfa Aesar, 98+%), pentaerythritol tetraacrylate (PETA, Sigma Aldrich), piperidine (Alfa Aesar, 99%), potassium carbonate (Bernd Kraft) and sodium hydrogencarbonate (J.T. Baker) were used as received.

Methods

The molar masses and the molar mass distribution ($Đ$) of the synthesized poly(2-oxazoline)s were measured using a PSS system (Agilent 1260) equipped with an autosampler, RI detector and an UV-detector type Agilent VWD Series 1260. The absorption was measured at $\lambda = 280$ nm. A so-called Gram Linear M column, equipped with a 10 µm particle size

precolumn was utilized at $T \approx 60$ °C. As eluent dimethylacetamide (DMAc) mixed with 1 g l^{-1} LiBr was used and 20 µl of the polymer-samples were injected. The flow rate of the system was 1 ml min^{-1}. The calibration curve was measured using PMMA standards (PSS, Mainz). Other gel permeation chromatography (SEC) experiments were carried out on an Agilent 1200-System consisting of a degasser, an isocratic pump, an autosampler, a RI-detector, a UV-detector (Lambda 1010, Bischoff), and a SDV Linear M column. THF was used as eluent with a flow rate of 1 ml min^{-1}. Calibration was performed with polystyrene standards (PSS, Mainz). The UV/Vis measurements were performed using an Evolution 220 UV-Visible spectrophotometer and a PCCU 1 Peltier control and cooling unit from Thermo Scientific. NMR spectroscopy was performed using a Bruker AV 400 spectrometer or a Joel ECZ 500 spectrometer. All measurements were performed at room temperature. The ^{1}H NMR spectra were recorded at 400 or 500 MHz and ^{13}C NMR spectra at 101 or 126 MHz. Chemical shifts (δ) are given in ppm and are referenced to the undeuterated signal of the used solvent. Polymerizations were performed using a Discover SP Microwave System equipped with an Explorer 12 Hybrid Autosampler from CEM. Crosslinking and photodeprotection experiments were carried out in a UV-crosslinker UVP-CL1000, operating at $\lambda = 365$ nm ($H_e = 12.0$ J cm^{-2} per 1 h).

<u>Synthesis of 2-{2-[(2-Nitrobenzyl)thio]ethyl}-4,5-dihydrooxazole (NbMEtOx)</u>

<u>A: Synthesis of 3-[(2-Nitrobenzyl)thio]propanoic acid (1)</u>

3-Mercaptopropionic acid (1.98 g, 18.7 mmol, 1.62 ml) and anhydrous NEt_3 (1.44 g, 14.2 mmol, 1.98 ml) were stirred in anhydrous acetone (60 ml) for 30 min at 0 °C. Then 2-nitrobenzyl bromide (2.02 g, 9.4 mmol) was added in portions under nitrogen. The reaction was stirred overnight at room temperature. The formed triethylamine hydrobromide was filtered-off and the crude product was isolated via rotary evaporation. The crude product was redissolved in dichloromethane (60 ml), washed with 0.5 M HCl (4 x 20 ml) and brine (20 ml). The organic

phase was dried over $MgSO_4$, filtrated and the product was concentrated via rotary evaporation. The obtained yellowish, highly viscous residue was stirred with hexanes (60 ml) for 30 min to obtain a yellowish-white powder as product. The title compound was filtered-off, washed with small portions of cold hexanes and dried in vacuo. TLC: R_f: 0 – 0.32 (EtOAc : hexanes 2:1) - Yield: 2.08 g (8.62 mmol, 92%) - ^{1}H NMR (400 MHz, $CDCl_3$) δ 10.90 (s, 1H), 7.98 (dd, 1H, J = 8.1, 1.3 Hz,), 7.57 (td, 1H, J = 7.5, 1.3 Hz), 7.51 – 7.39 (m, 2H), 4.10 (s, 2H), 2.75 – 2.69 (m, 2H), 2.65 – 2.59 (m, 2H) ppm; ^{13}C NMR (101 MHz, $CDCl_3$) δ 177.7, 148.9, 134.0, 133.3, 132.0, 128.5, 125.6, 34.4, 33.8, 26.7 ppm.

B: Synthesis of 2,5-Dioxopyrrolidin-1-yl 3-[(2-nitrobenzyl)thio]propanoate (2)

3-((2-Nitrobenzyl)thio)propanoic acid (2.08 g, 8.62 mmol), NEt_3 (1.43 g, 14.1 mmol, 2.0 ml) and NHS (1.62 g, 14.1 mmol) were dissolved in DCM (60 ml). To the yellowish mixture, EDC-HCl (2.70 g, 14.1 mmol) was added and the mixture was stirred at room temperature overnight. The mixture was washed with 1 M HCl (3 x 40 ml), water (3 x 40 ml) and brine (2 x 40 ml). The aqueous phases were combined and extracted with DCM (30 ml). The solvent was evaporated and the crude product was directly used for preparation of compound **3**.

C: Synthesis of N-(2-chloroethyl)-3-[(2-nitrobenzyl)thio]propenamide (3)

Compound **2** (2.98 g, 8.8 mmol) was dissolved in DCM (30 ml) and cooled to 0 °C. To this solution 2-chloroethylamine hydrochloride (2.05 g, 17.6 mmol) and NEt_3 (1.78 g, 17.6 mmol, 2.5 ml) were added and the mixture was stirred for 45 min at 0 °C. Afterwards, the cooling bath was removed and the solution was stirred until TLC (EtOAc : hexanes 4:1, R_f: 0.65 [product **3**]) showed complete conversion (around 2 h). The mixture was diluted with DCM (30 ml) and 1 M HCl (20 ml) was added. The organic phase was further washed with 1 M HCl (2 x 20 ml), brine (20 ml) and saturated aq. $NaHCO_3$ (3 x 20ml) The organic phase was dried with $MgSO_4$, filtrated and the solvent was evaporated under reduced pressure. The product was isolated as yellow-white solid. Yield: 2.34 g (7.7 mmol, 89%) - ^{1}H NMR (400 MHz, $CDCl_3$) δ 7.96 (dd, 1H, J = 8.1, 1.3 Hz), 7.56 (td, 1H, J = 7.5, 1.3 Hz), 7.49 (dd, 1H, J = 7.7, 1.4 Hz), 7.45 – 7.39 (m, 1H), 6.09 (br.s, 1H), 4.09 (s, 2H), 3.64 – 3.56 (m, 4H), 2.76 (t, 2H, J = 7.1 Hz), 2.44 (t, 2H, J = 7.1 Hz) ppm. ^{13}C NMR (101 MHz, $CDCl_3$) δ 171.3, 148.8, 134.0, 133.3, 132.2, 128.5, 125.5, 44.0, 41.4, 36.5, 33.8, 27.8 ppm.

D: Ring formation to 2-{2-[(2-Nitrobenzyl)thio]ethyl}-4,5-dihydrooxazole (NbMEtOxa) (4)

Amide **3** (2.31 g, 7.7 mmol) was dissolved in MeCN (50 ml). Anhydrous K_2CO_3 (2.74 g, 19.9 mmol) was added to the solution and stirred at 80 °C until TLC (EtOAc, R_f: 0.20 [product **4**])

showed full conversion (around 16 h). After cooling the reaction suspension, the solids were decanted and then passed through a syringe filter (PTFE, 0.45 μm). Then, the solvent was evaporated and the product was dried in vacuum overnight. The obtained yellow oil was dissolved in DCM, passed through a syringe filter (PTFE, 0.45 μm), and the solvent was removed in vacuo. Afterwards the crude product was passed through a short silica column (eluent: EtOAc) and then the solvent was evaporated. The product appeared as light brownish solid after drying in vacuum overnight. Yield: 1.22 g (4.57 mmol, 59%) - ^{1}H NMR (400 MHz, $CDCl_3$) δ 7.96 (dd, 1H, J = 8.1, 1.3 Hz), 7.55 (td, 1H, J = 7.5, 1.3 Hz), 7.48 (dd, 1H, J = 7.7, 1.5 Hz), 7.44 – 7.37 (m, 1H), 4.21 (t, 2H, J = 9.5 Hz), 4.09 (s, 2H), 3.81 (t, 2H, J = 9.5 Hz), 2.76 – 2.70 (m, 2H), 2.55 – 2.48 (m, 2H) ppm. ^{13}C NMR (101 MHz, $CDCl_3$) δ 166.6, 148.8, 134.1, 133.2, 132.0, 128.3, 125.5, 67.5, 54.5, 33.7, 28.5, 28.2 ppm.

Synthesis of CH_3-Poly[(2-ethyl-2-Oxazoline)$_{1-x}$-co-(NbMEtOx)$_x$]-piperidine (Px%)

All polymerizations were performed according to the following procedure. As example, the polymerization using 10 mol% NbMEtOxa is described.

Briefly, MeOTf (5.61 mg, 34 μmol) and EtOxa (10.17 mg, 100 μmol) were dissolved in anhydrous MeCN (1091 mg) in a glovebox. Then the mixture was stirred under microwave irradiation for 15 min at 120 °C. Afterwards, the solution was transferred into a glovebox, in which EtOxa (384 mg, 3.88 mmol) and NbMEtOxa (103.3 mg, 0.38 mmol) were added to the vial. The final concentration was adjusted to c = 2 mol l^{-1} by adding anhydrous MeCN. Then, the mixture was stirred under microwave irradiation for 90 min at 120 °C. The polymerization was stopped by adding 1 M methanolic piperidine solution (0.2 ml) and the resulting mixture was stirred at room temperature overnight. The polymer was precipitated in ice-cold diethyl ether, redissolved in 1,4-dioxane, filtrated and freeze dried. Yield: 370 mg (74%) - ^{1}H NMR (500 MHz, $CDCl_3$) δ 8.02 – 7.87 (8H), 7.62 – 7.35 (25H), 4.15 – 4.03 (17H), 3.66 – 3.27 (401H), 3.06 – 2.98 (3H), 2.80 – 2.58 (26H), 2.48 – 2.12 (192H), 1.21 – 0.89 (292H) ppm.

Deprotection Procedure

The corresponding copolymer (100 mg, 6.7 µmol) was filled into a microwave vial and dissolved in MeCN (30 ml). The vial was sealed with a silicone cap and the mixture was purged with Ar for 15 min. Afterwards, the vial was placed in a UV crosslinking chamber with a wavelength of λ = 365 nm for 2 h (H_e = 24 J cm^{-2}). Then the polymer was recovered by precipitation into ice-cold diethyl ether. The precipitate was collected and thoroughly washed with diethyl ether. Finally, the product was dried in vacuum overnight.

Kinetics of Photodeprotection

Solutions of different polymer concentrations were prepared in MeCN. Each solution was added to a 1 cm square quartz cell. The cells were irradiated at λ = 365 nm in a UV crosslinking chamber (H_e = 12 J cm^{-2} per 1 h). After 5 min, the irradiation was stopped and the cells immediately transferred to a UV spectrometer, where the absorbance was recorded at λ = 310 nm.

Gelformation using Pentaerythritol tetraacrylate (PETA)

The polymer **P10%** containing protected thiol groups (50 mg, 3.3 µmol, 36.3 µmol thiol groups) and PETA (0.7 equiv. with respect to thiol groups) were dissolved in degassed DMF to obtain a concentration of 15 wt%. Then HMPP (2.7×10^{-3} mg, 1.64×10^{-2} µmol, 2.5 µl) was added. The vial was sealed and remaining oxygen was removed by a freeze-pump-thaw cycle. Afterwards, the vial was placed in a UV crosslinker (λ = 365 nm, H_e = 12 J cm^{-2}) for 1 h.

Attempts to synthesize a NbMEtOxa Homopolymer

MeOTf and MeOxa were dissolved in anhydrous MeCN and stirred for 15 minutes in the microwave oven at 120 °C with an initial power of 140 W. Then, NbMEtOxa was added in a glovebox to the reaction mixture. The reaction vial was transferred back to the microwave oven

and further stirred for 90 min (120°C, initial power 140 W). Subsequently, the polymerization was quenched by adding water (0.2 ml) and stirred for 4 hours at room temperature. The polymer was precipitated into ice-cold diethyl ether, dissolved in 1,4-dioxane and dried via lyophilization. The individual reaction parameters are listed in the following table.

Table S1: Tabular representation of the initial weights of MeOTf, MeCN, MeOxa, NbMEtOxa, yields, and GPC analysis data for NbMEtOxa homopolymers.

No.	MeOTf /mg (µmol)	MeCN /g	MeOxa /mg (µmol)	NbMEtOxa /g (mmol)	Yield /mg (%)	$\bar{M}_n$ /kDa	$Đ$
1	3.1 (19)	1.2	3.8 (45)	0.20 (0.77)	- [a]	- [a]	- [a]
2	4.4 (27)	1.8	5.1 (60)	0.27 (1.0)	- [a]	- [a]	- [a]
3	2.9 (17)	2.3	6.2 (73)	0.19 (0.72)	20 (10)	1.3	2.9

[a] Homopolymer could not be isolated.

NMR Spectra of NbMEtOxa and Precursors

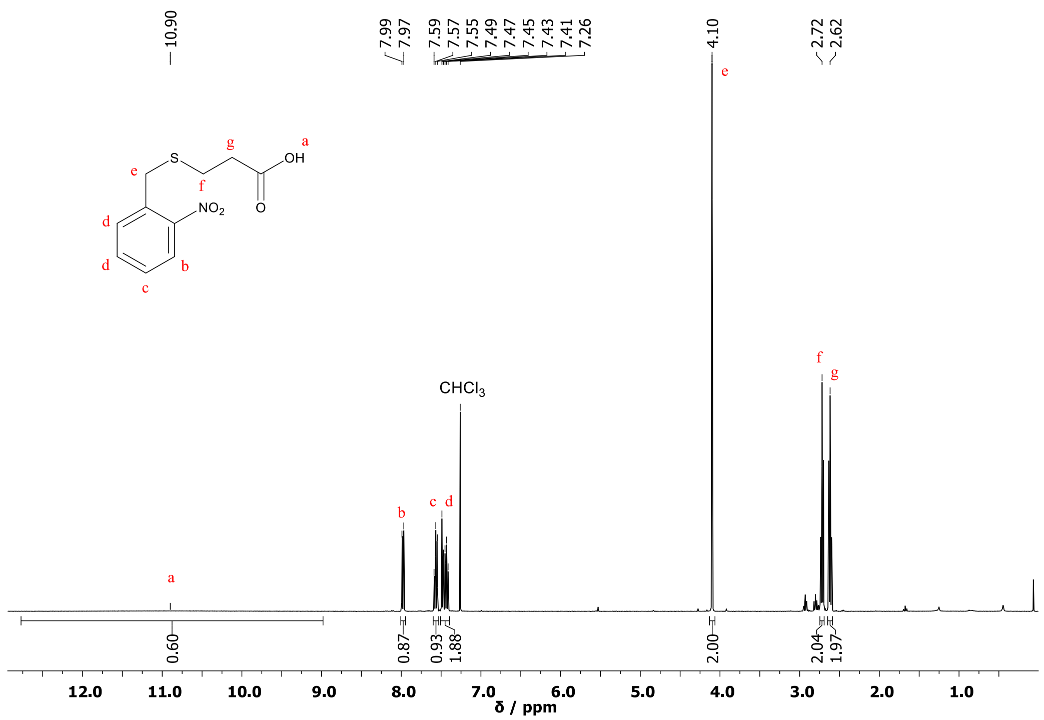

Figure S1: ^{1}H NMR (400 MHz) spectrum of 3-[(2-nitrobenzyl)thio]propanoic acid (**1**) recorded in $CDCl_3$.

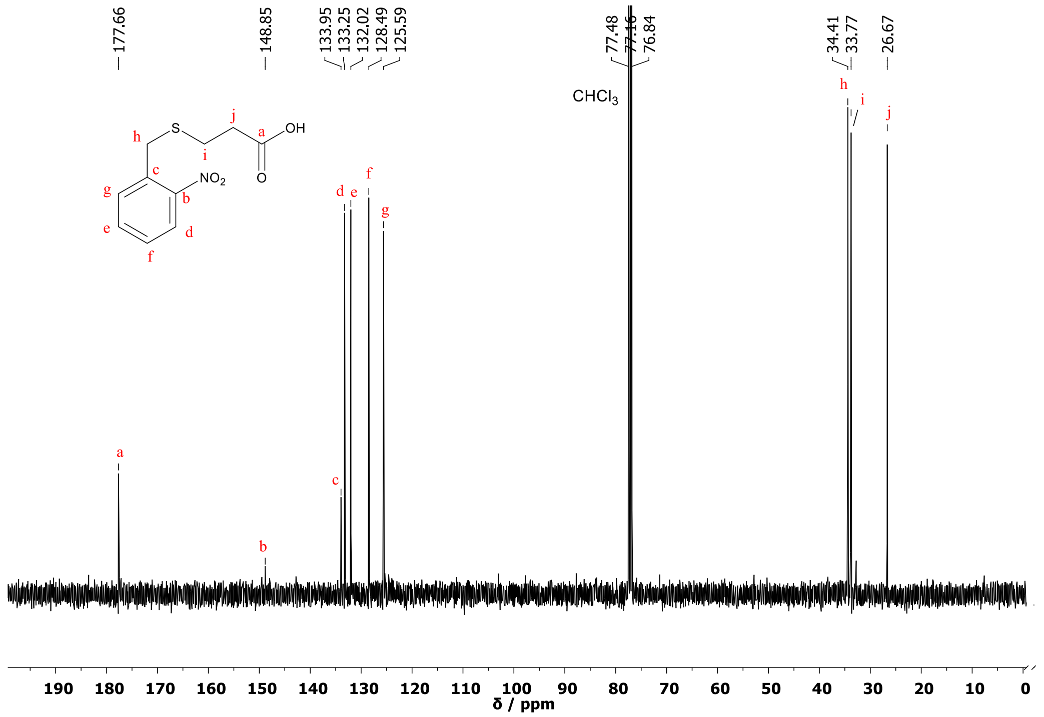

Figure S2: ^{13}C NMR (101 MHz) spectrum of 3-[(2-nitrobenzyl)thio]propanoic acid (**1**) recorded in $CDCl_3$.

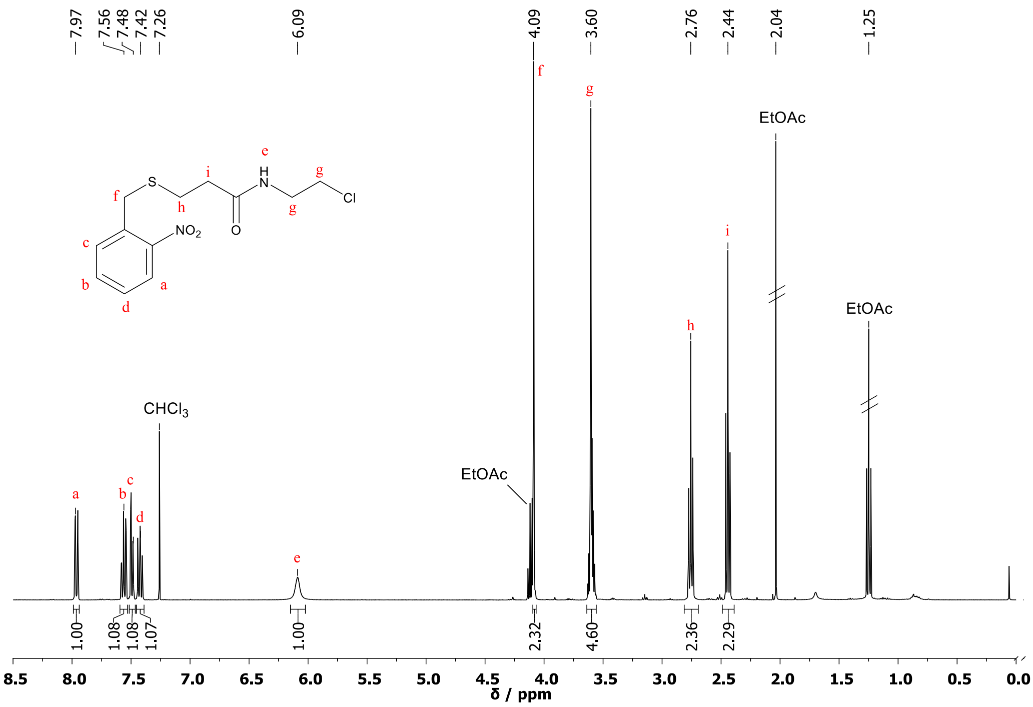

Figure S3: ^{1}H NMR (400 MHz) spectrum of *N*-(2-chloroethyl)-3-[(2-nitrobenzyl)thio]propenamide (**3**) recorded in $CDCl_3$.

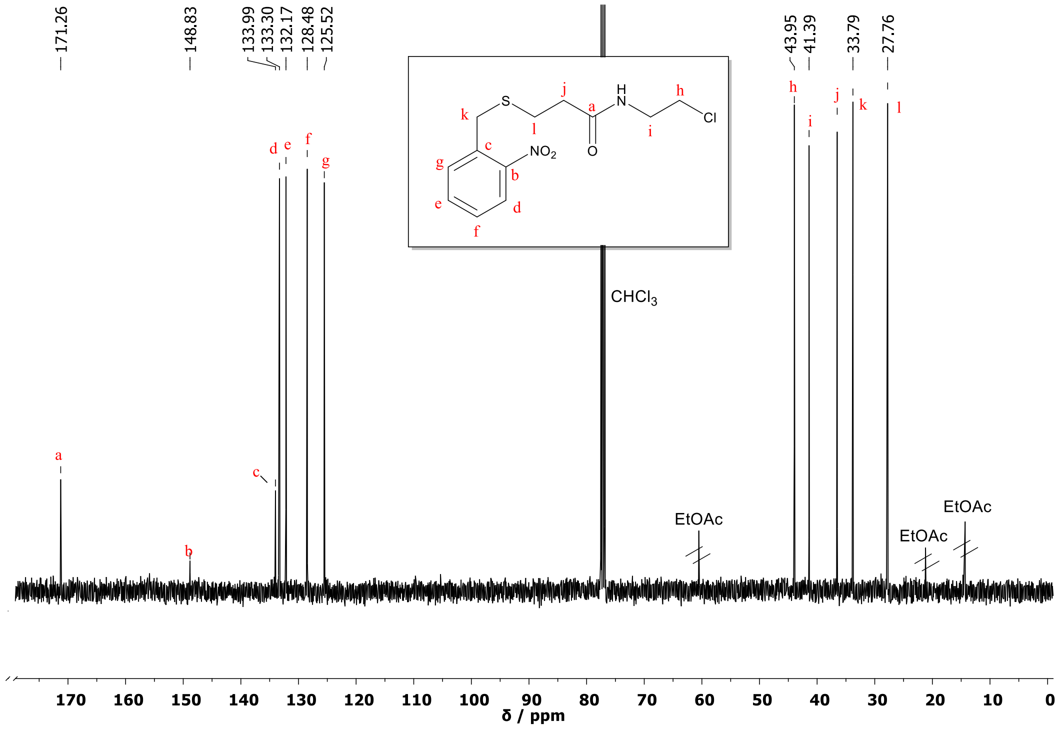

Figure S4: ^{13}C NMR (101 MHz) spectrum of *N*-(2-chloroethyl)-3-[(2-nitrobenzyl)thio]propenamide (**3**) recorded in $CDCl_3$.

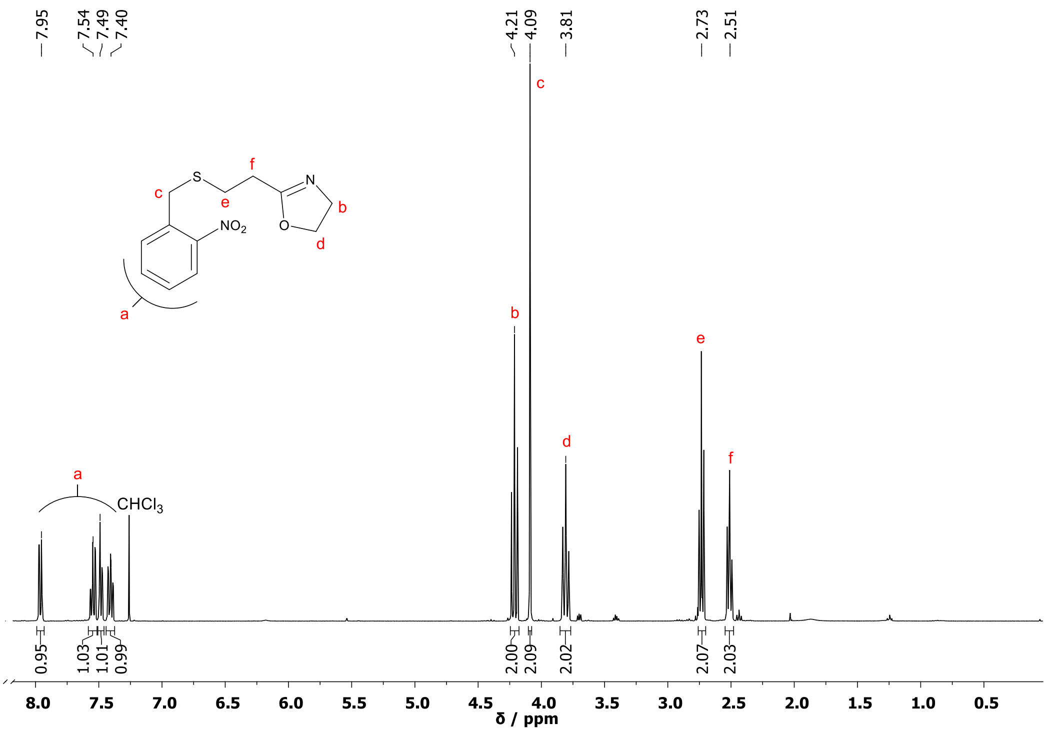

Figure S5: ^{1}H NMR (400 MHz) spectrum of 2-{2-[(2-nitrobenzyl)thio]ethyl}-4,5-dihydrooxazole (NbMEtOx) (**4**) recorded in $CDCl_3$.

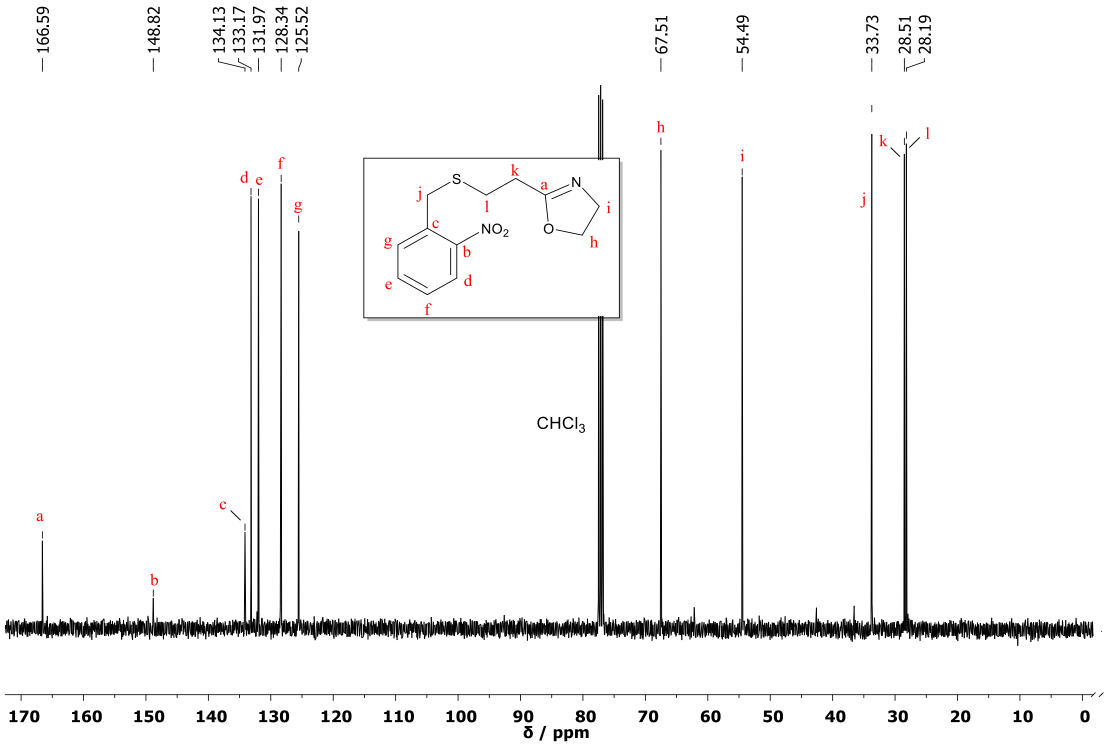

Figure S6: ^{13}C NMR (101 MHz) spectrum of 2-{2-[(2-nitrobenzyl)thio]ethyl}-4,5-dihydrooxazole (NbMEtOx) (**4**) recorded in $CDCl_3$.

NMR Spectra of Polymers before and after Deprotection

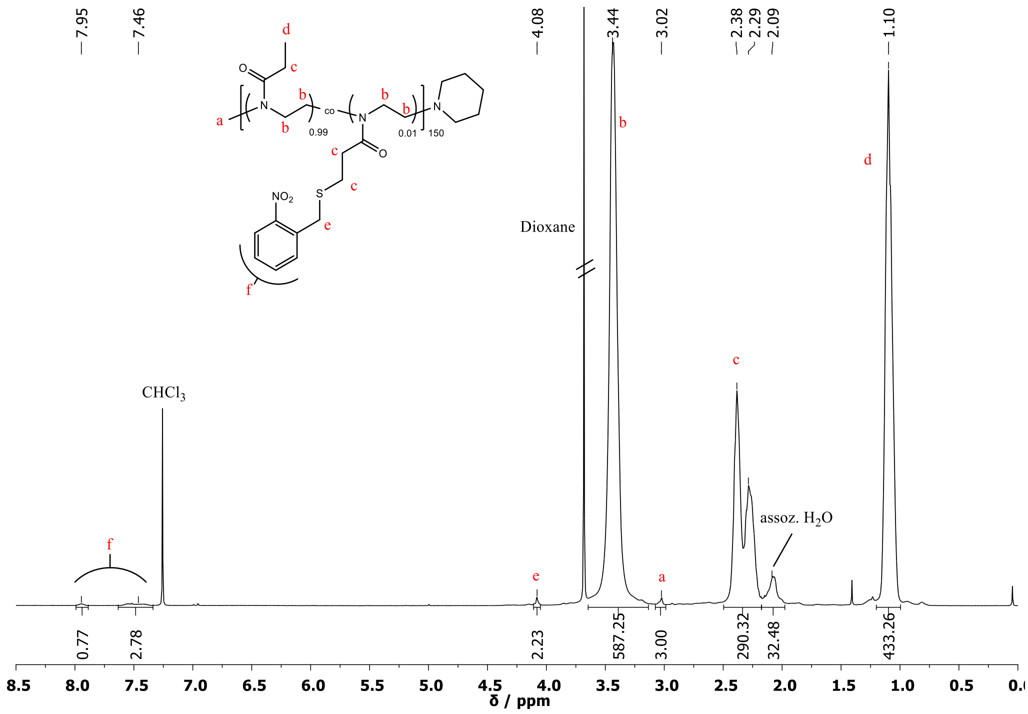

Figure S7: ^{1}H NMR (500 MHz) spectrum of **P1%** recorded in $CDCl_3$.

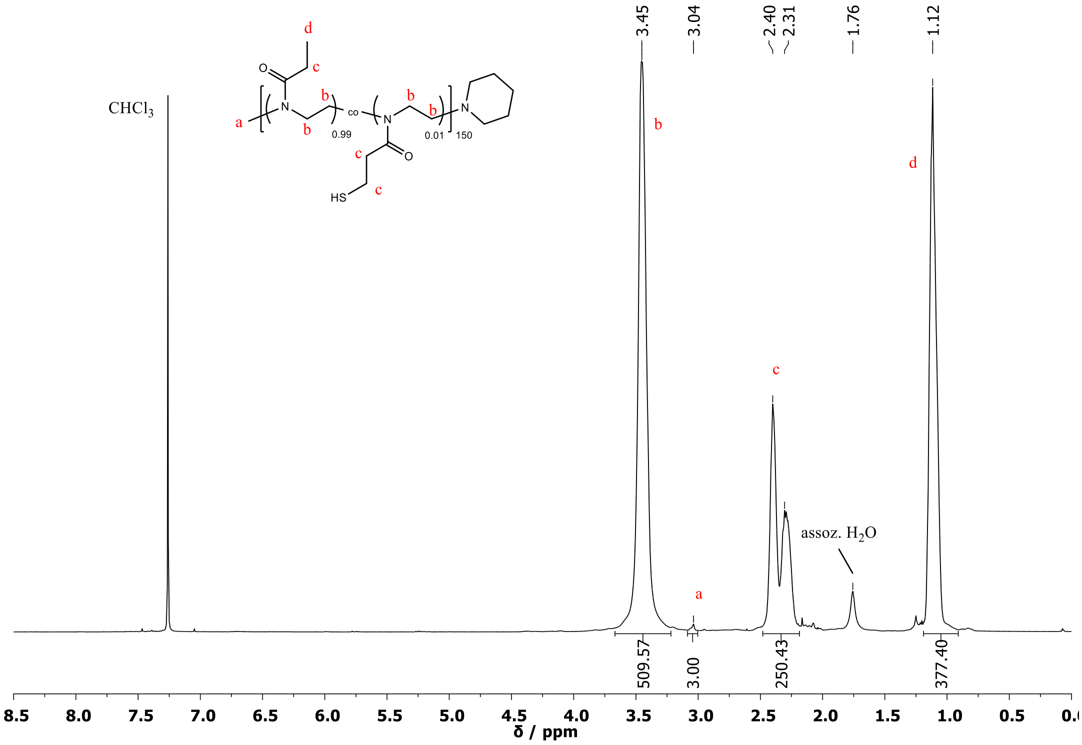

Figure S8: ^{1}H NMR (500 MHz) spectrum of **P1%** after deprotection recorded in $CDCl_3$.

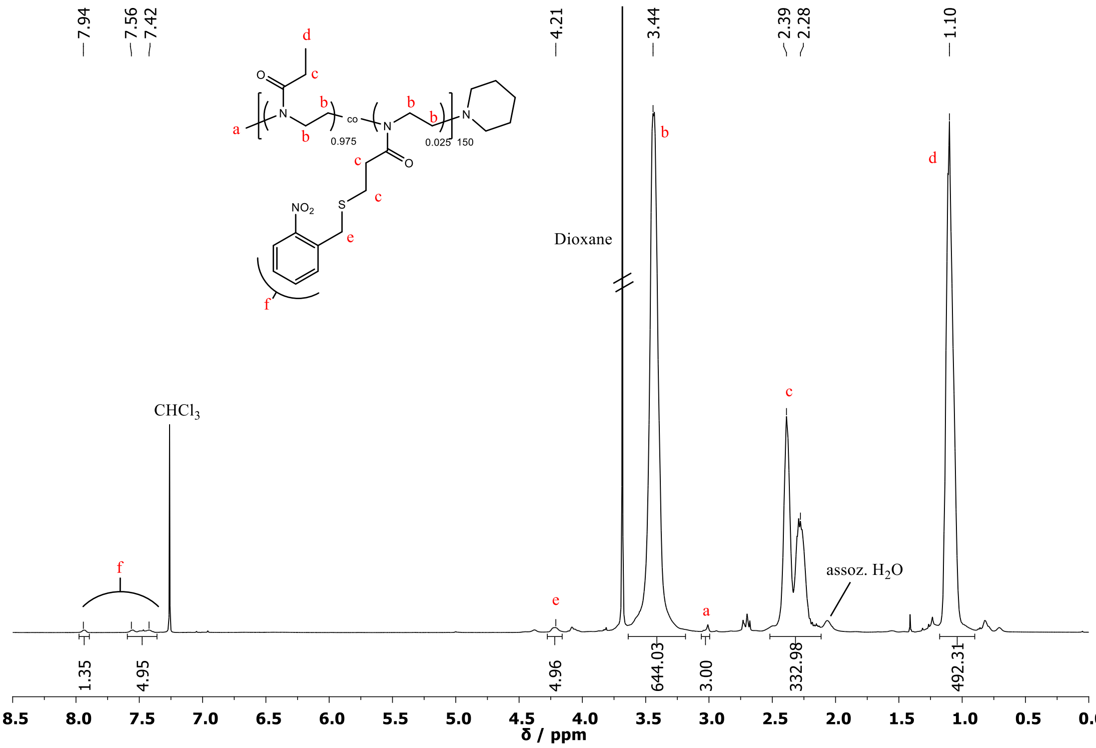

Figure S9: ^{1}H NMR (500 MHz) spectrum of **P2.5%** recorded in $CDCl_3$.

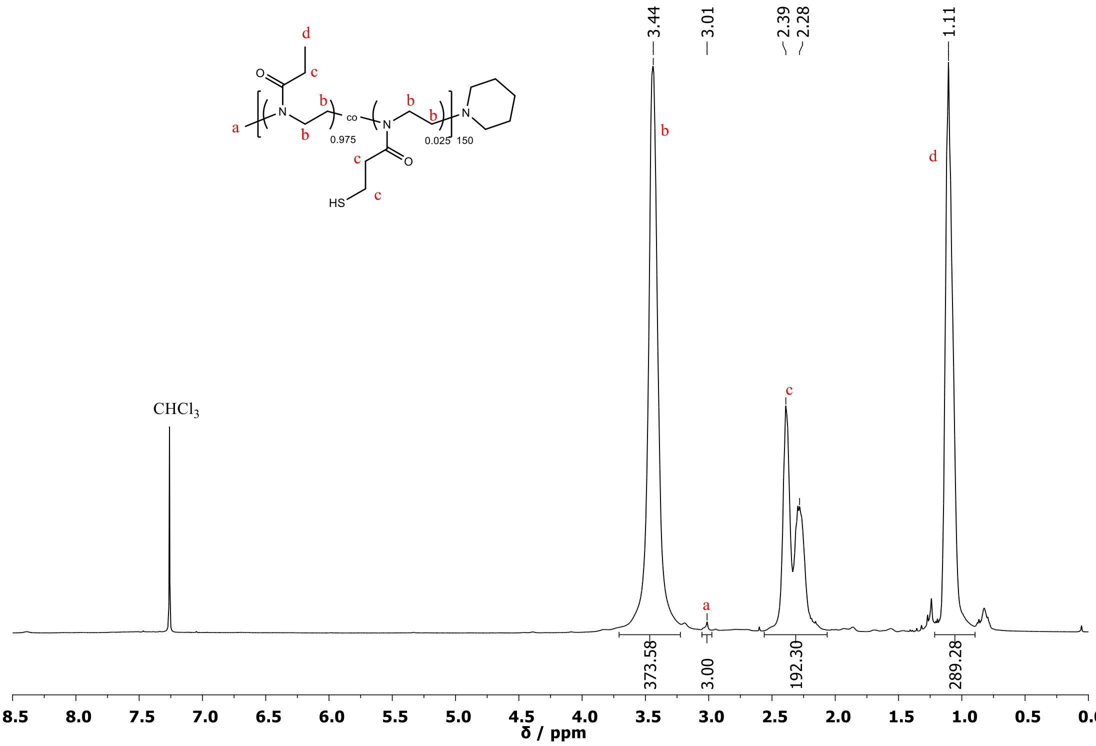

Figure S10: ^{1}H NMR (500 MHz) spectrum of **P2.5%** after deprotection, recorded in $CDCl_3$.

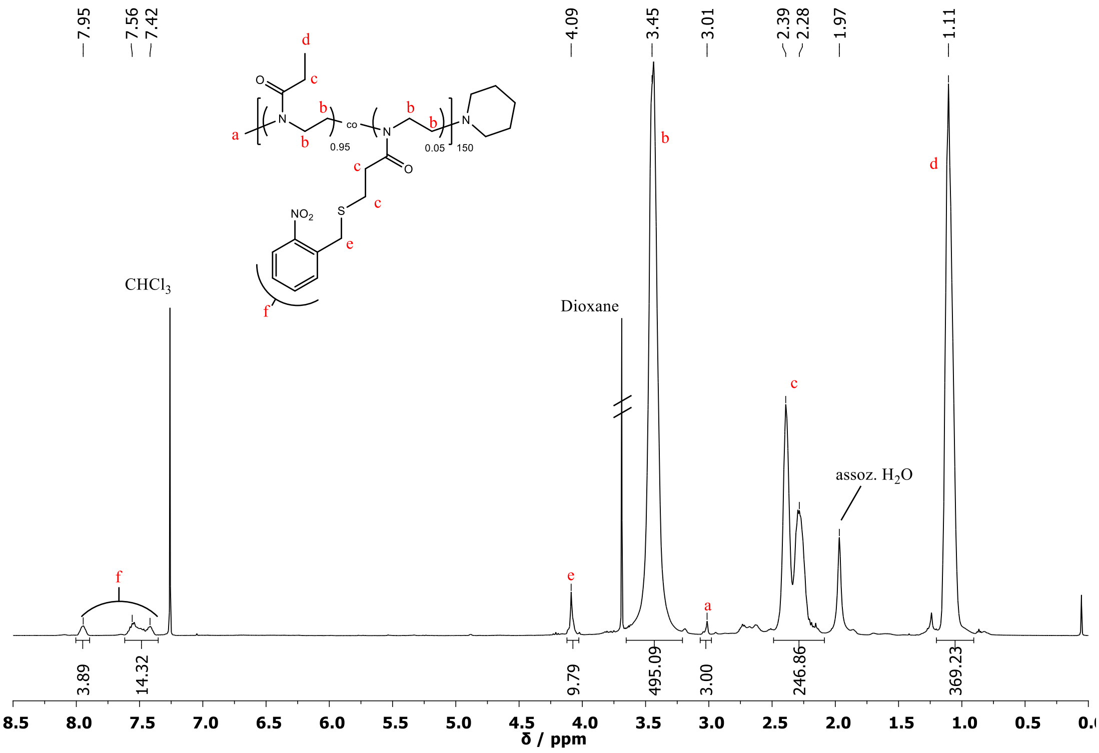

Figure S11: ^{1}H NMR (500 MHz) spectrum of **P5%** recorded in $CDCl_3$.

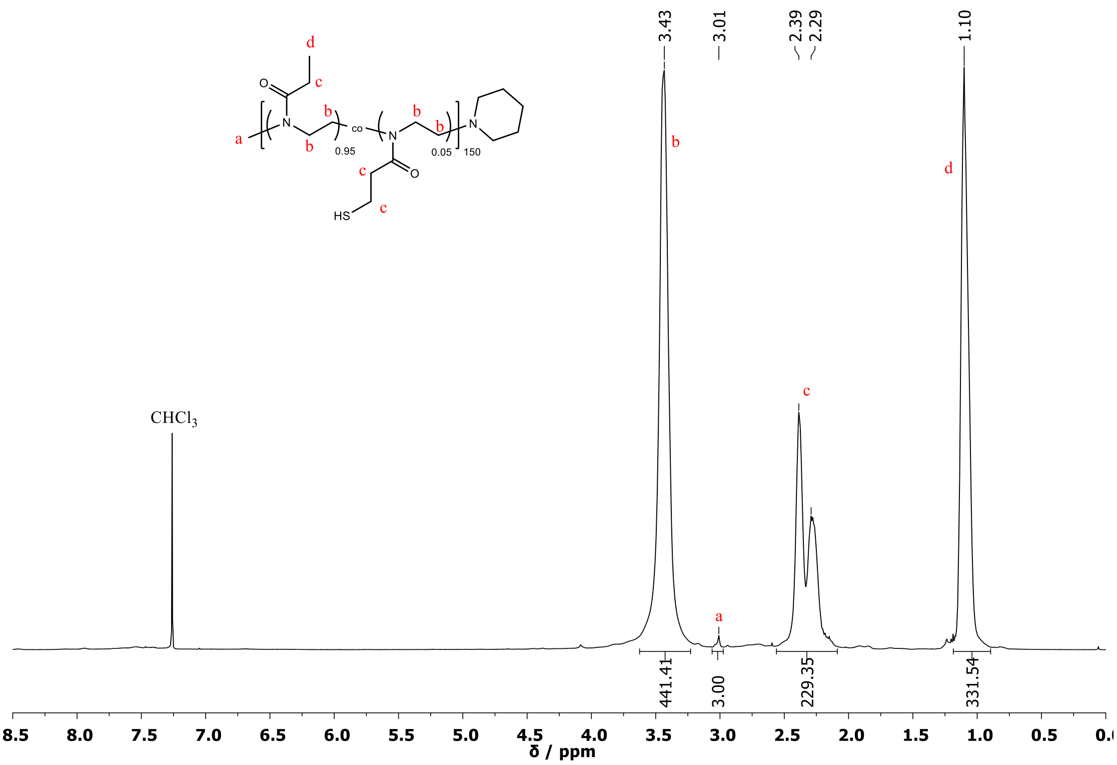

Figure S12: ^{1}H NMR (500 MHz) spectrum of **P5%** after deprotection, recorded in $CDCl_3$.

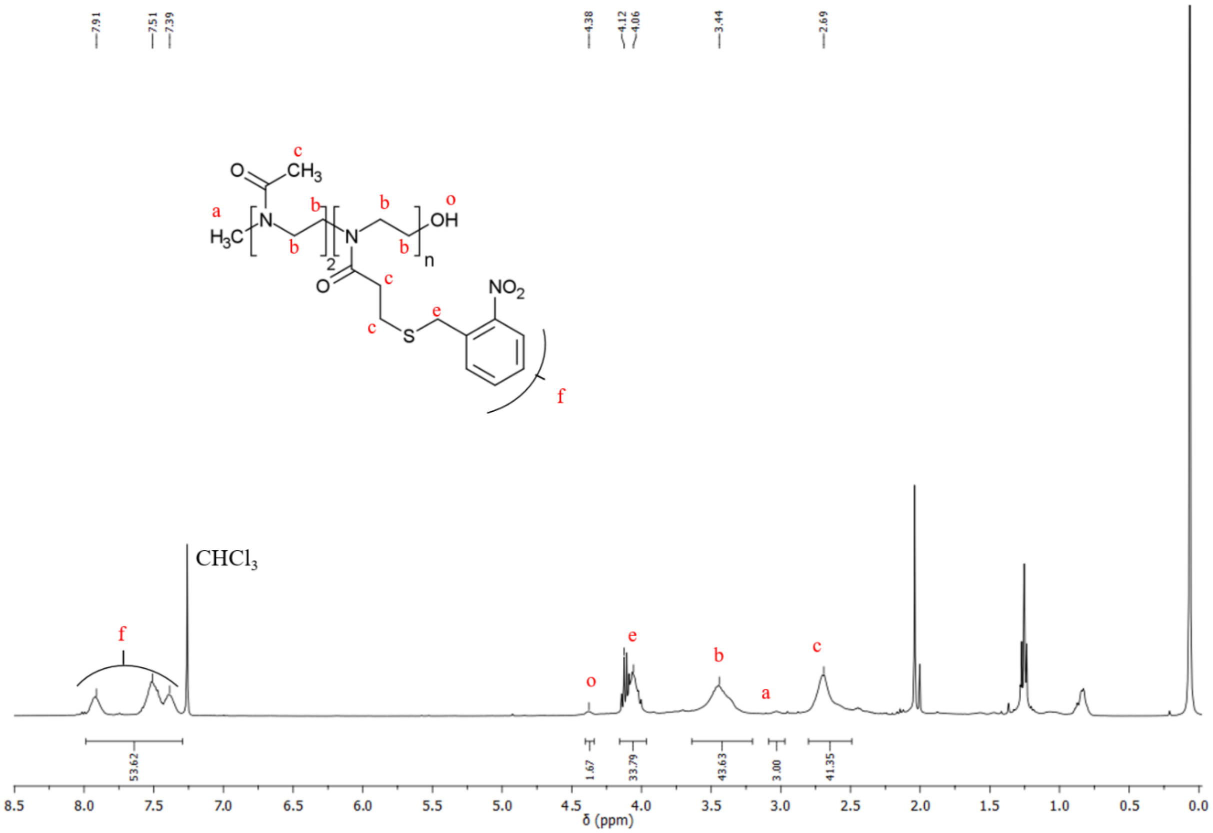

Figure S13: ^{1}H NMR (400 MHz) spectrum of the NbMEtOxa homopolymer, recorded in $CDCl_3$.

GPC Data of Polymers

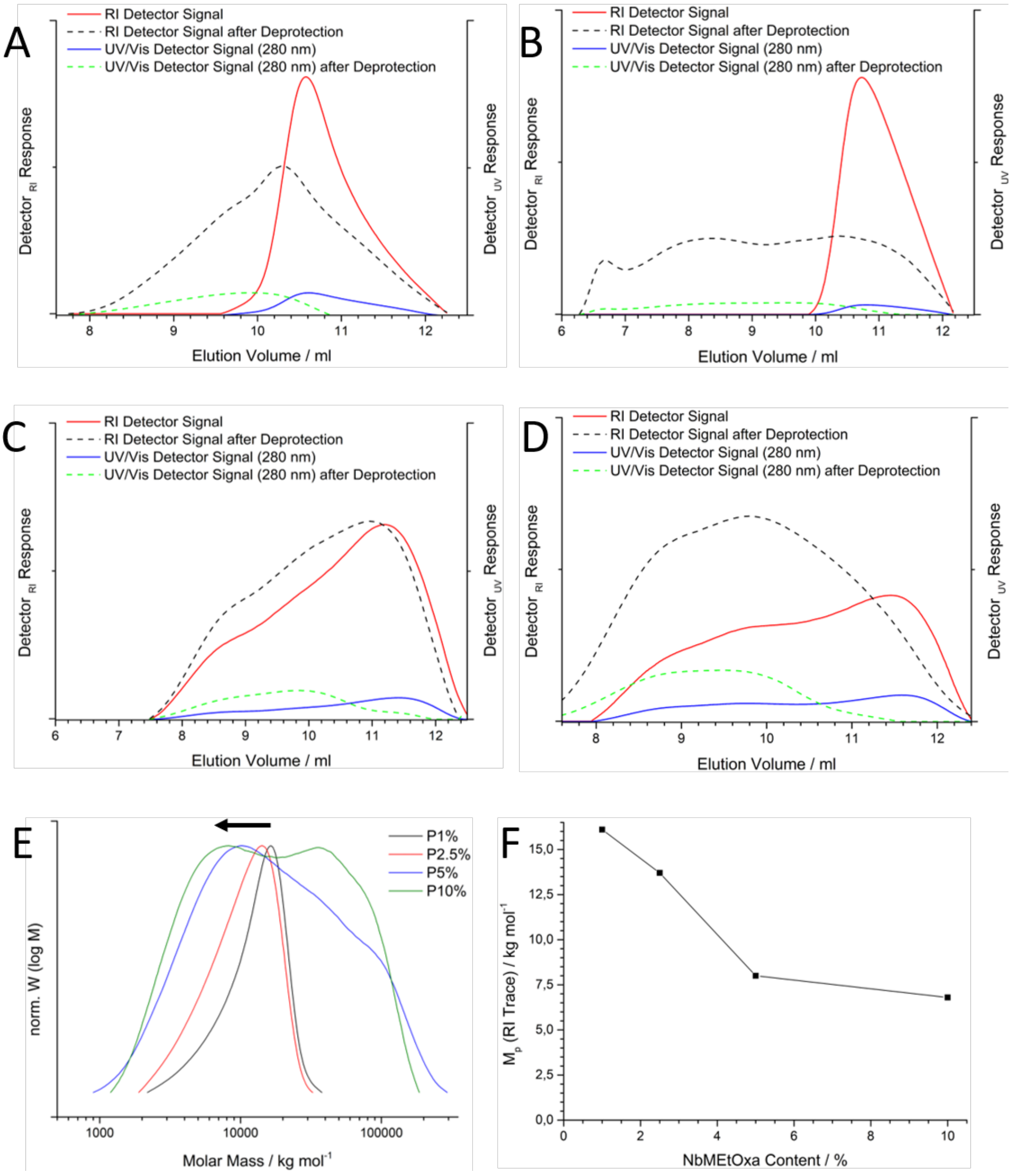

Figure S14: GPC elugrams of the different copolymers containing NbMEtOxa. The content of NbMEtOxa increased in the order from **P1%** (A), **P2.5%** (B), **P5%** (C) to **P10%** (D). DMAc containing LiBr (1 g l^{-1}) was used as eluent for each measurement. In graph (E), the RI GPC traces for all copolymers (**P1%**, **P2.5%**, **P5%**, **P10%**) are plotted to illustrate the change in molar mass at peak maximum (M_p), as indicated in panel (F).

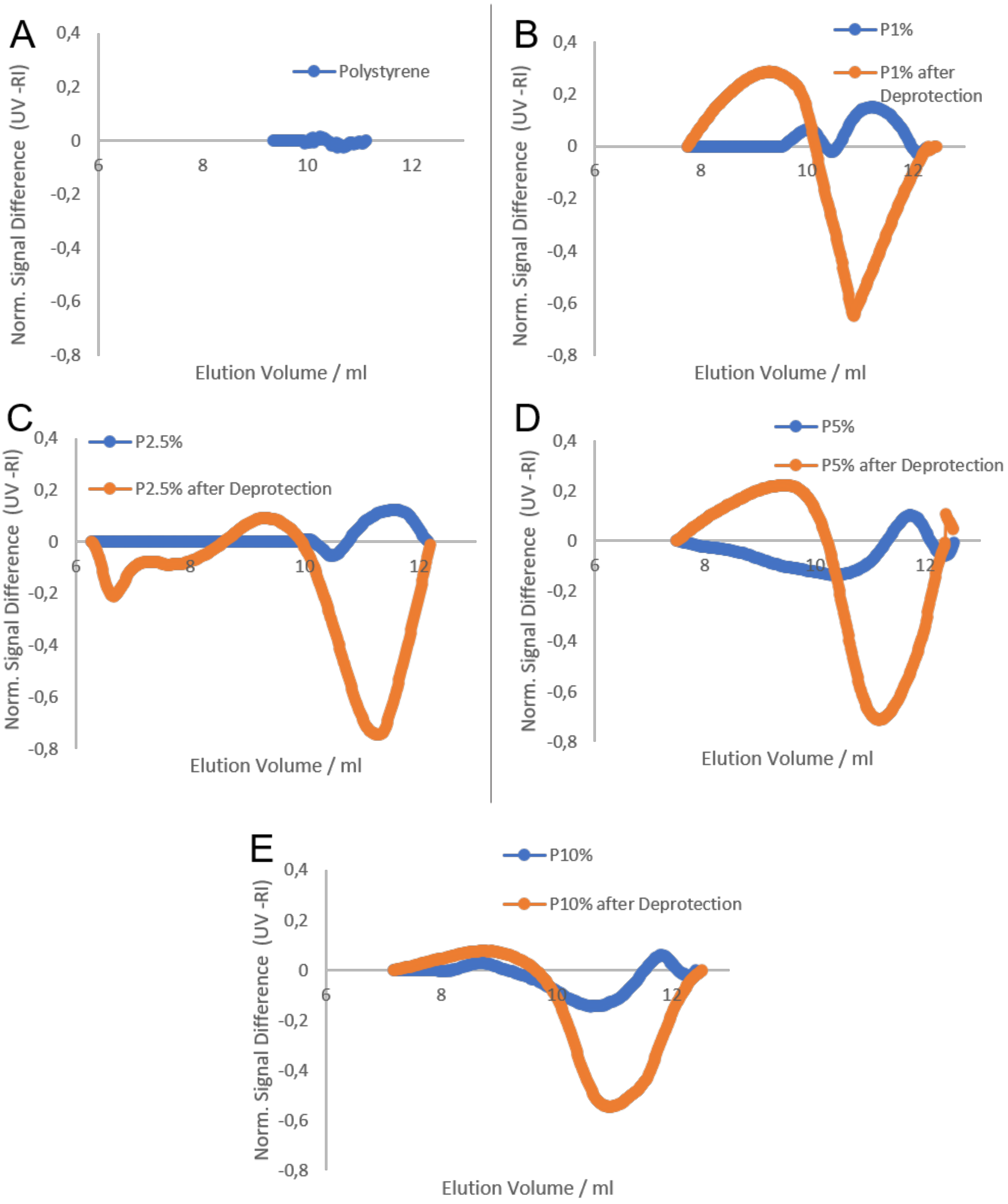

Figure S15: Qualitative analysis of the compositional drift by UV-RI difference plots for a polystyrene (PS) standard 5630 g mol^{-1} (A), **P1%** (B), **P2.5%** (C), **P5%** (D) to **P10%** (E) based on the following procedure: (1) first the UV and RI traces were normalized by dividing all signal intensity values with the one at the respective trace maxima and (2) then the normalized RI trace was subtracted from the normalized UV trace. The reference trace (A) shows a close to zero-value line as expected for the PS homopolymer. The blue lines show the copolymer systems before and the orange lines after irradiation with UV light (λ = 365 nm, H_e = 12 J cm^{-2}, 1 h).

UV/Vis Spectra of Polymers

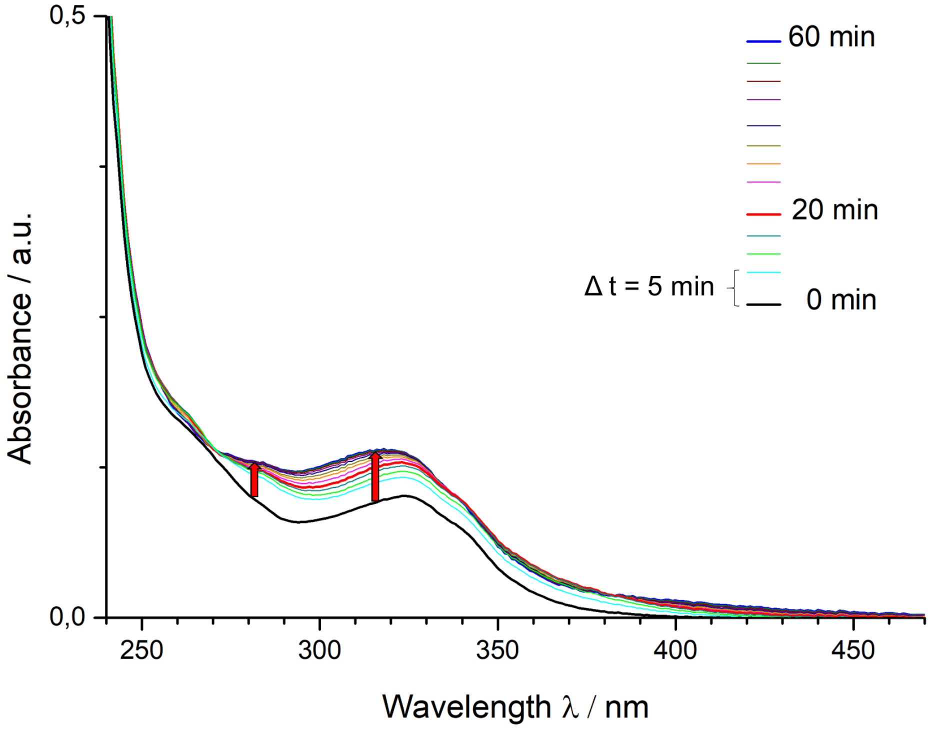

Figure S16: Time-dependent UV/Vis absorbance vs. wavelength scans of **P1%**. Red arrows indicate the change in absorption with increasing time.

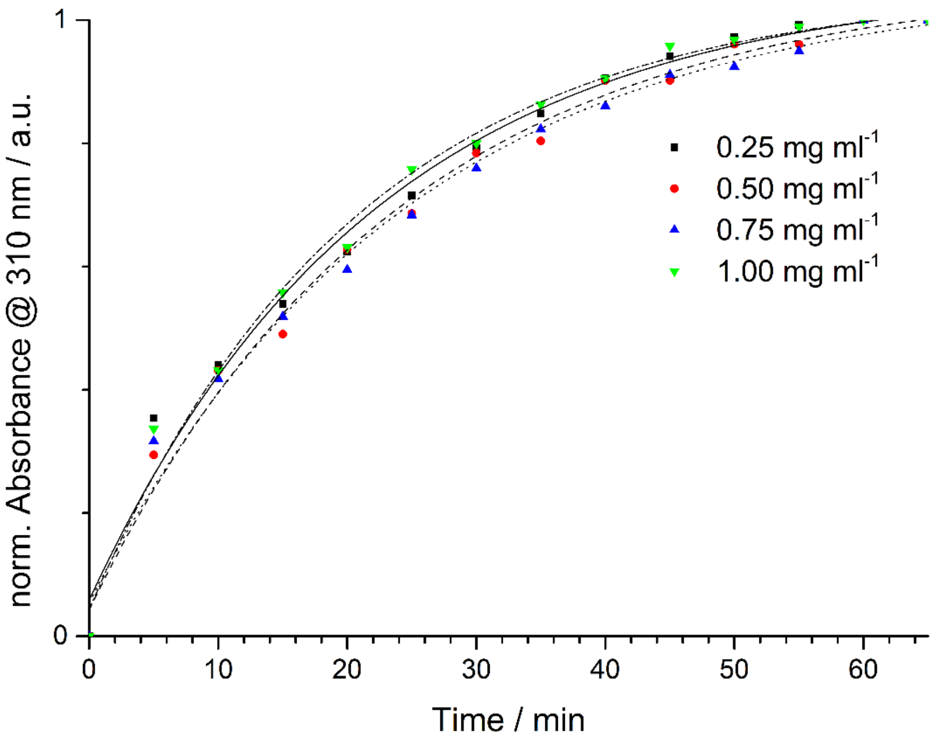

Figure S17: Deprotection kinetics of **P1%** at different concentrations. The absorbance was measured at λ = 310 nm.

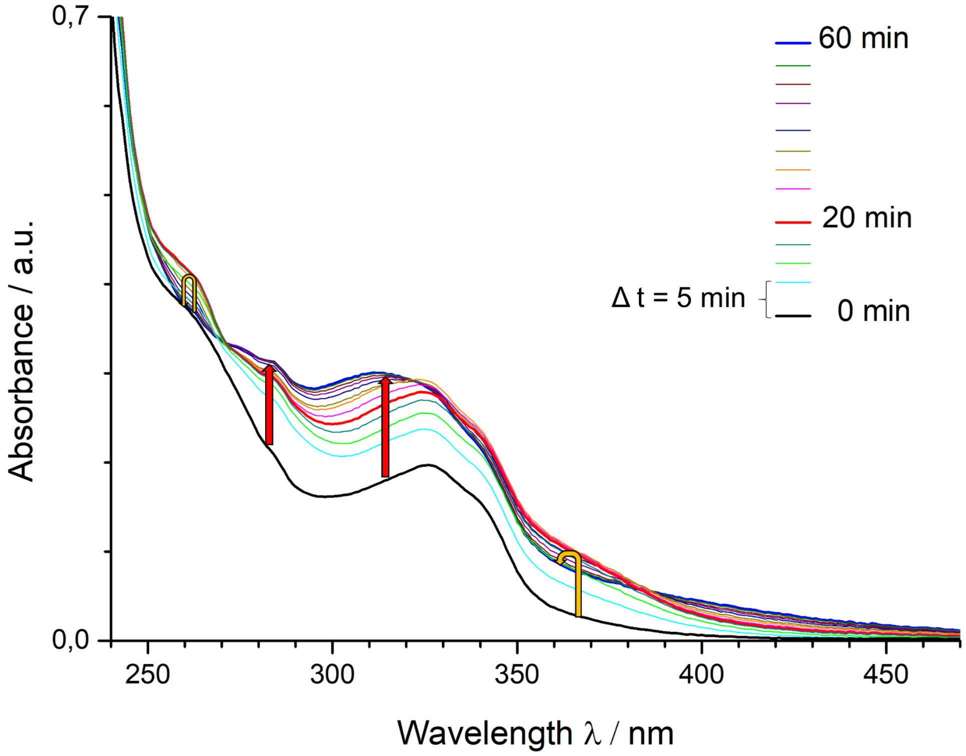

Figure S18: Time-dependent UV/Vis absorbance vs. wavelength scans of **P2.5%**. Red and yellow arrows indicate the change in absorption with increasing time.

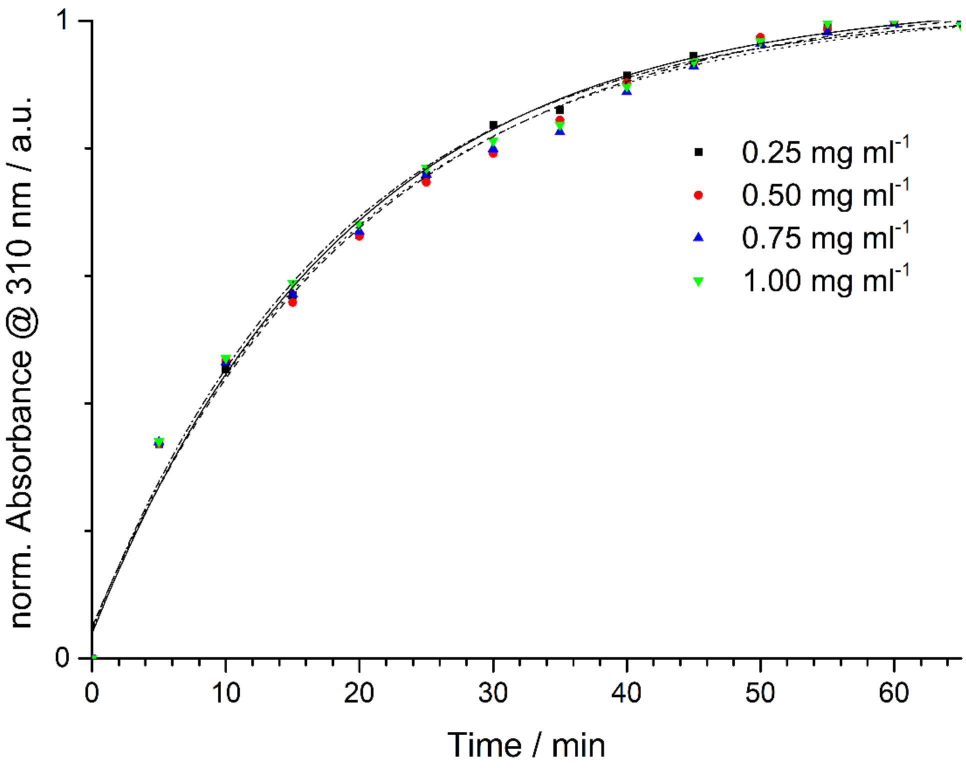

Figure S19: Deprotection kinetics of **P2.5%** at different concentrations. The absorbance was measured at λ = 310 nm.

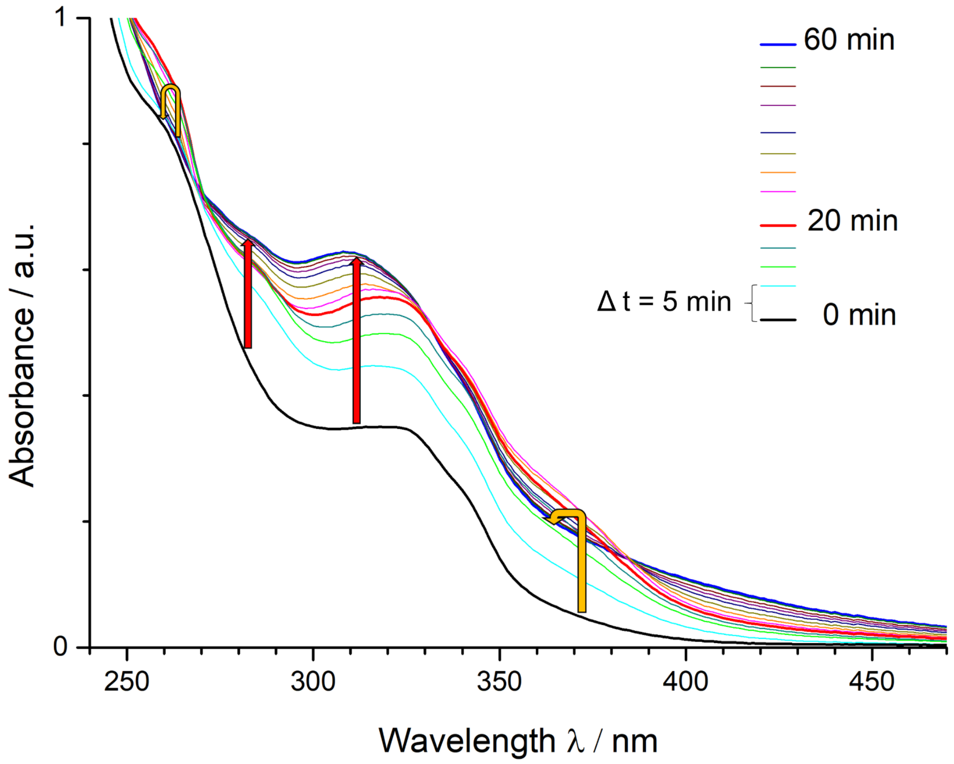

Figure S20: Time-dependent UV/Vis absorbance vs. wavelength scans of **P5%**. Red and yellow arrows indicate the change in absorption with increasing time.

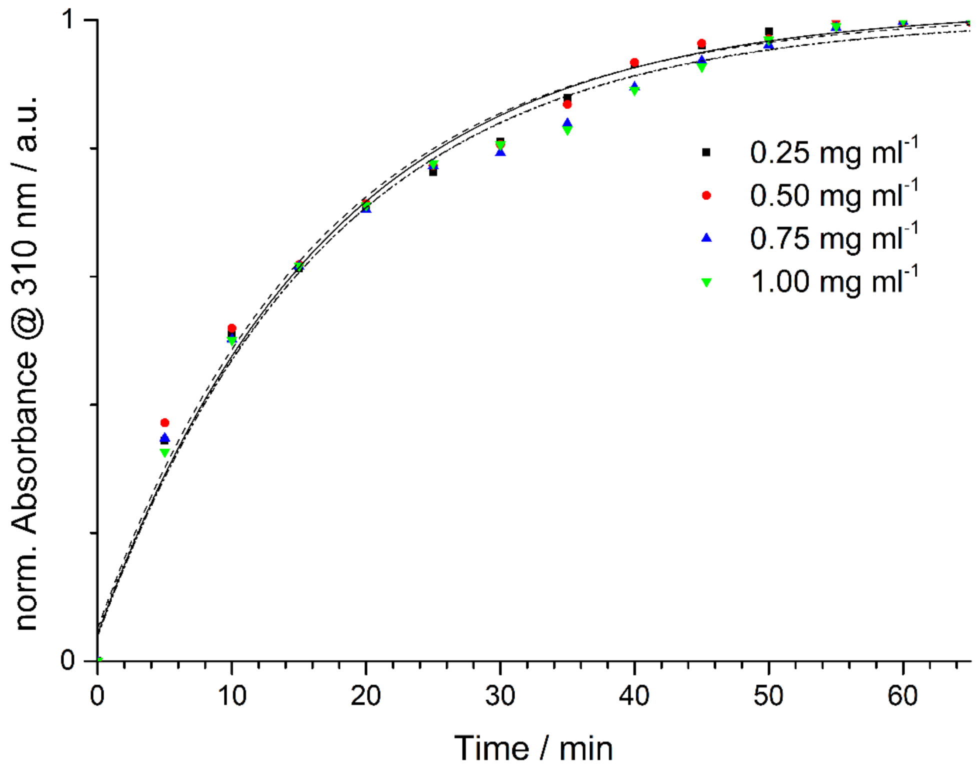

Figure S21*:* Deprotection kinetics of **P5%** at different concentrations. The absorbance was measured at λ = 310 nm.

Gel Formation

Table S2: Quantities of polymer, crosslinker PETA and DMF used for crosslinking tests. Photoinitiator HMPP (2.7 µg, 1.64×10^{-2} µmol, 2.5 µl) was added to reaction vials 2 to 6.

Vial	Polymer	Mass Polymer / mg (content of Thiols / µmol)	Mass PETA[a] / mg (µmol, µl)	Total Volume DMF / µl
**1**	P10%	50 (36.3)	-	331
**2**	-	-	2.3 (25.4, 21.5)	350
**3**	P1%	50 (3.4)	0.21 (2.4, 2.0)	348
**4**	P2.5%	50 (10.4)	0.65 (7.3, 6.1)	344
**5**	P5%	50 (18.1)	1.15 (12.7, 10.7)	340
**6**	P10%	50 (36.3)	2.3 (25.4, 21.5)	331

[a] Mass of PETA was taken from a freshly prepared stock solution in DMF (β = 107 mg ml^{-1}).

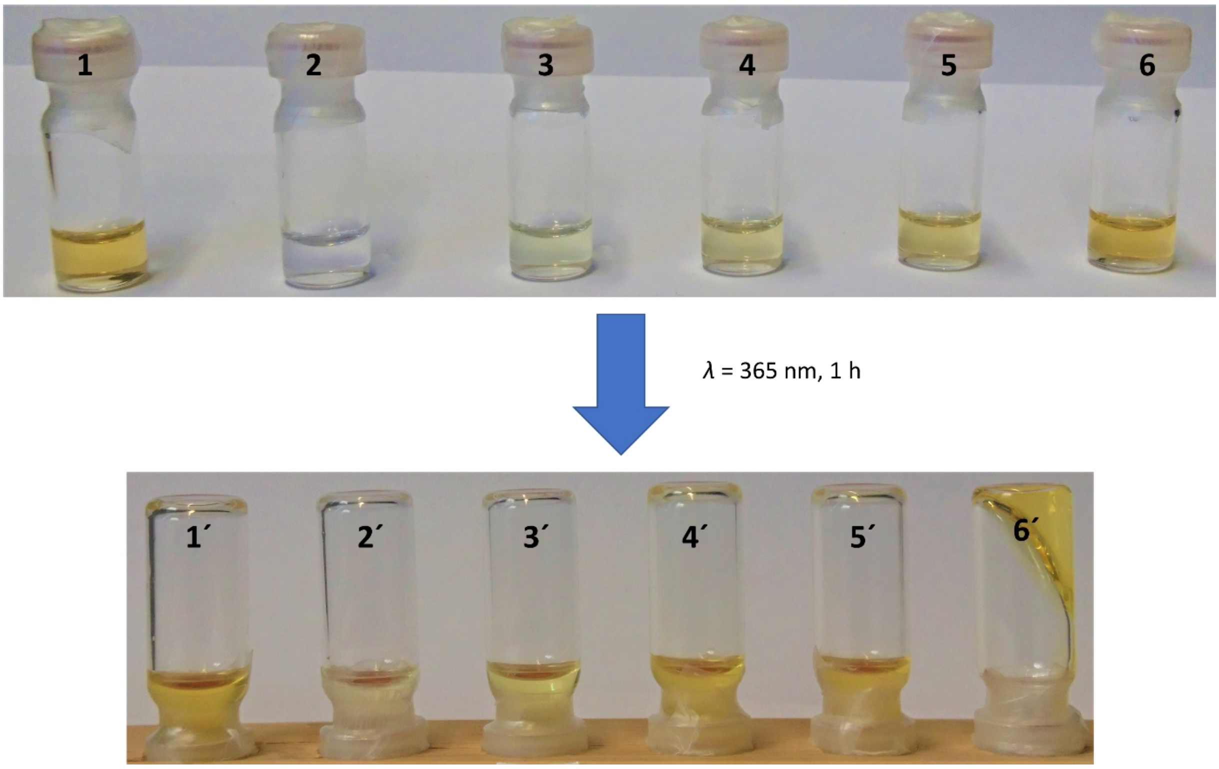

Figure S22: Images of reaction vials before (upper picture) and after irradiation (lower picture, vial numbers are marked with a prime). The vial numbers correspond to numbering in Table S2. The concentration of polymer was 15 wt% in DMF. Vials **1** and **2** were used as control. Successful network formation in vial **6’** is corroborated by the triangular shape of the rigid gel after irradiation of the tilted vial.
